# Rapid glycation with D-ribose induces globular amyloid-like aggregations of BSA with high cytotoxicity to SH-SY5Y cells

**DOI:** 10.1186/1471-2121-10-10

**Published:** 2009-02-13

**Authors:** Yan Wei, Lan Chen, Ji Chen, Lin Ge, Rong Qiao He

**Affiliations:** 1 State Key Laboratory of Brain and Cognitive Sciences, Institute of Biophysics, Chinese Academy of Sciences, 15 Da Tun Road, Chaoyang District, Beijing, 100101, PR China; 2 Graduate University of Chinese Academy of Sciences, 19A Yu Quan Road, Shijingshan District, Beijing, 100039, PR China

## Abstract

**Background:**

D-ribose in cells and human serum participates in glycation of proteins resulting in advanced glycation end products (AGEs) that affect cell metabolism and induce cell death. However, the mechanism by which D-ribose-glycated proteins induce cell death is still unclear.

**Results:**

Here, we incubated D-ribose with bovine serum albumin (BSA) and observed changes in the intensity of fluorescence at 410 nm and 425 nm to monitor the formation of D-ribose-glycated BSA. Comparing glycation of BSA with xylose (a control for furanose), glucose and fructose (controls for pyranose), the rate of glycation with D-ribose was the most rapid. Protein intrinsic fluorescence (335 nm), Nitroblue tetrazolium (NBT) assays and Western blotting with anti-AGEs showed that glycation of BSA incubated with D-ribose occurred faster than for the other reducing sugars. Protein intrinsic fluorescence showed marked conformational changes when BSA was incubated with D-ribose. Importantly, observations with atomic force microscopy showed that D-ribose-glycated BSA appeared in globular polymers. Furthermore, a fluorescent assay with Thioflavin T (ThT) showed a remarkable increase in fluorescence at 485 nm in the presence of D-ribose-glycated BSA. However, ThT fluorescence did not show the same marked increase in the presence of xylose or glucose. This suggests that glycation with D-ribose induced BSA to aggregate into globular amyloid-like deposits. As observed by Hoechst 33258 staining, 3-(4, 5-dimethylthiazol-2-yl)-2,5-diphenyl tetrazolium bromide (MTT) and cell counting kit-8 (CCK-8) assay, lactate dehydrogenase (LDH) activity assay, flow cytometry using Annexin V and Propidium Iodide staining and reactive oxygen species (ROS) measurements, the amyloid-like aggregation of glycated BSA induced apoptosis in the neurotypic cell line SH-SY5Y.

**Conclusion:**

Glycation with D-ribose induces BSA to misfold rapidly and form globular amyloid-like aggregations which play an important role in cytotoxicity to neural cells.

## Background

Non-enzymatic glycation of proteins by reducing saccharides such as glucose (glc) and ribose (rib) leads to the formation of fructosamine [1] and advanced glycation end products (AGEs) [2,3]. Among the reducing monosaccharides, the role of glc in the glycation of proteins has been widely studied, and is implicated in diabetes [4], cataracts [5], renal failure [6], and other disorders [7]. It has recently become clear that glycation is also involved in physiological neurodegenerative diseases such as Alzheimer's disease [8]. Glycation alters the biological activity of proteins and their degradation processes. Protein cross-linking by glycation results in the formation of detergent-insoluble and protease-resistant aggregates. Therefore, the study of AGEs has become one of the most important areas of biomedical research today.

The glc-glycation process that gives rise to AGEs requires a long time. Many reports indicate that glycation with glc advances slowly and is associated with every fundamental process in cellular metabolism [9-11]. Although a great deal of work has been carried out on glycation of proteins with glc, few research groups have attempted to investigate the role of rib in glycation and its resulting effects on cell metabolism. Compared with glc, rib is more active in glycation of proteins and the formation of AGEs [12,13].

The role of D-rib in glycation and cross-linking of collagen has been investigated in *in vitro *studies of the triggering of skin ageing [14]. Luciano and colleagues (2008) prepared proteins glycated with rib in a study of AGEs and their effects on pancreatic islet beta-cells. Direct effects of AGEs on cellular viability and related insulin secretion of beta-cells resulting from their exposure to glycated serum by incubation with rib have been evaluated. Results showed reduced cellular proliferation with a corresponding increase in cell necrosis and cell apoptosis rate in comparison with untreated cells after 5 days of exposure to glycation conditions [15].

Further studies have shown that glycation reactions lead to the production of reactive oxygen species (ROS), which are harmful to cellular metabolism and cause cell damage [16]. Consequently, considerable attention has been given in recent years to studying the generation of hydroxyl radicals from Amadori proteins, and inhibition of hydroxyl radical damage [17]. As with glc-glycation, glycation of proteins with rib also involves Amadori rearrangement and then production of AGEs. Therefore, some research groups have employed glycation with rib instead of glc as a model for investigating the mechanism by which AGEs yield hydroxyl radicals and induce cell apoptosis or necrosis [15,17,18].

Most recently, formation of molten globule-like states has been reported during the progression of glycation reactions *in vitro *[19]. According to Dobson et al., globule-like protein aggregations (pro-amyloid fibrils) are significantly toxic to neurons [20-23]. However, the characteristics and cytotoxicity of molten globule-like protein states induced by glycation have not been clarified. There are no reports in the literature showing that glycation with rib can induce protein misfolding and aggregation in amyloid-like deposits.

Glycation of serum albumin has been widely studied in recent years [1,15,24,25], and bovine serum albumin (BSA) is commonly used as molecular model. Significantly, Friedman and colleagues have reported neurotoxicity for brain-penetrant serum albumin including BSA [26,27]. This study is concerned with determining whether glycation of BSA with D-rib in the short term (1–7 days) results in globular amyloid-like aggregations that are seriously toxic to neuroblast cells through apoptosis, compared with other pyranose and furanose.

## Results

### BSA is rapidly glycated in the presence of ribose

**Figure 1:**
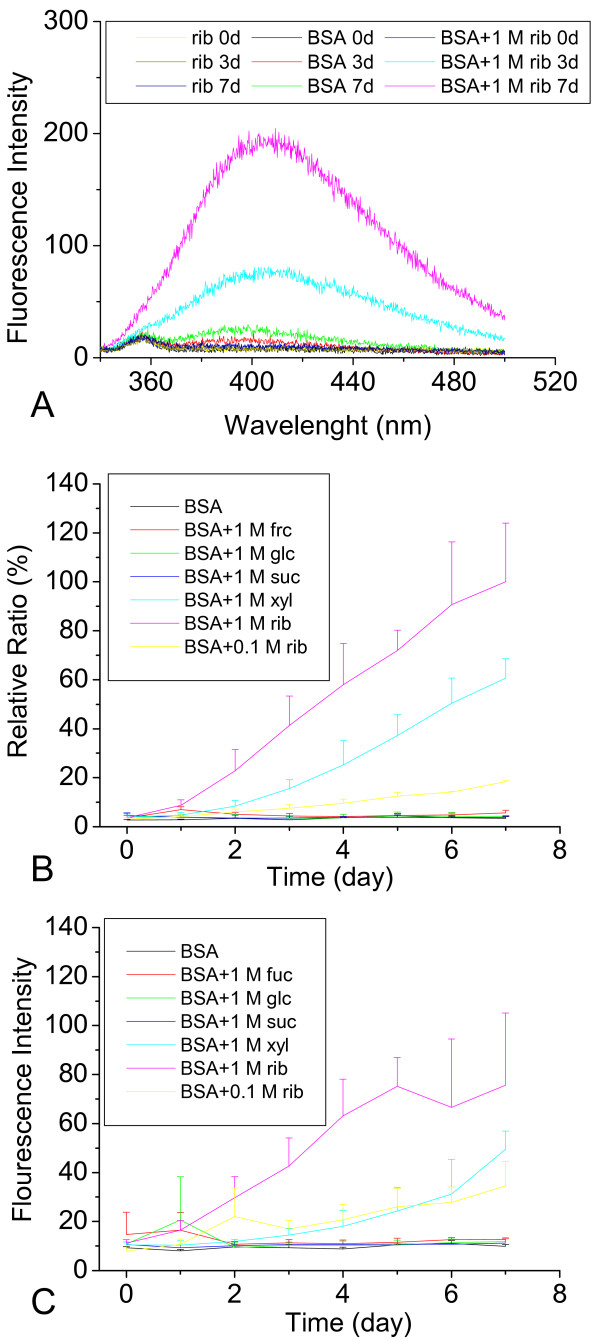
**Time course of changes in the fluorescence of BSA incubated with different saccharides**. BSA (final concentration 150 μM) in the presence of D-ribose (rib, final concentration 1 M) was kept at 37°C in Tris-HCl buffer (pH 7.4). BSA or rib alone was used as a control. (A) Aliquots were taken for measurements of fluorescence spectra (λ_ex _= 320 nm) at different time intervals. (B) Time course of changes in the maximal fluorescent intensity (λ_ex_320 nm; λ_em_410 nm) of BSA incubated with rib, glucose (glc), fructose (frc), xylose (xyl) and sucrose (suc). (C) Changes in fluorescence at λ_ex _370 nm; λ_em _425 nm were also detected under the same conditions.

To investigate whether glycation of a protein with rib is a rapid process, we incubated BSA with rib, glc, xylose (xyl), fructose (frc) or sucrose (non-reducing sugar, suc), and monitored changes in the intensity of the fluorescence (λ_ex _= 320 nm/λ_em _= 410 nm; λ_ex _= 370 nm/λ_em _= 425 nm) that is commonly used to detect the formation of glycated products [28-31]. Figure 1A illustrates changes in the relative emission intensities at 410 nm of BSA incubated with D-rib. Of the monosaccharides tested, the fluorescence of rib-glycated BSA increased most markedly with time and had the shortest relaxation time (about one day). The increase in the relative intensity (%) of the fluorescence for rib-glycated BSA was the fastest with an increase of 16.37% per day during the incubation period (Figure 1B, Table 1). The relative fluorescence intensity of xyl-glycated BSA as a control for furanose for 7 days was ~51% of that of rib-glycated BSA (indicated as 100%) and had a relatively short relaxation time. However, changes in the fluorescent emission of BSA in the presence of glc and frc as controls for pyranose were not significantly different under the experimental conditions used here. BSA alone, or in the presence of suc (non-reducing sugar) as a negative control, showed no significant changes in fluorescence. This suggests that furanose, especially rib, reacts much more rapidly with BSA than pyranose in the production of glycated products.

**Table 1:** Relaxation time of relative increase in intrinsic fluorescence intensity of BSA with different monosaccharides

	λ_ex _320 nm/λ_em _410 nm		λ_ex _280 nm/λ_em _335 nm
	
	Relaxation time (d)	Increase (%)	Decrease (%)
BSA	-	-	-

BSA+frc	-	-	-

BSA+glc	-	-	-

BSA+suc	-	-	-

BSA+xyl	2	8.4	5.89

BSA+rib	1	16.37	7.43

BSA+0.1M rib	3	2.48	1.98

Glycation of BSA in the presence of rib at low concentrations was also investigated (Figure 1B). A moderate increase in emission intensity at 410 nm of glycated BSA was observed in the presence of 0.1 M rib. This suggests that glycation efficiency of BSA depends on the rib concentration.

Similar results were observed when fluorescence at 425 nm was measured. As shown in Figure 1C, a rapid increase in the relative fluorescent intensity of rib-glycated BSA was also observed under the same conditions. This suggests again that rib is more active in protein glycation than the other reducing sugars used in this work.

**Figure 2:**
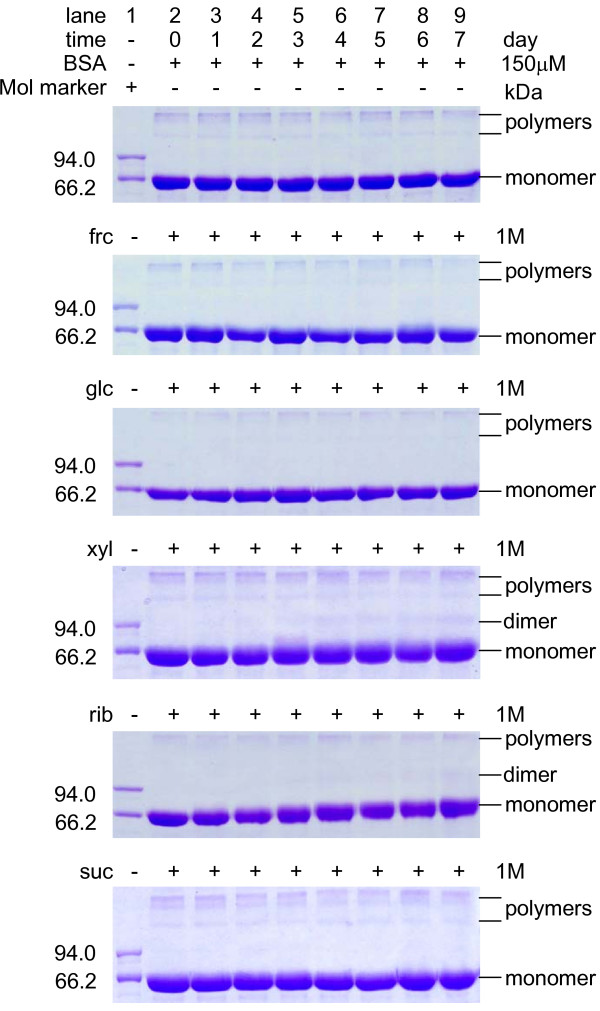
**10% SDS-PAGE of the products of BSA incubation with different saccharides**. Incubation conditions were as in Figure 1, except that aliquots were taken from incubation mixtures at different time intervals for SDS-PAGE electrophoresis.

To investigate further whether glycation was faster in the presence of rib than other reducing sugars, we incubated BSA with rib and analysed aliquots taken at different time intervals by SDS-PAGE (Figure 2). Retardation of protein bands was observed during the glycation of BSA in the presence of rib. The increasing apparent molecular masses of BSA during glycation probably resulted from bound rib. Analysis using mass spectrometry showed that the difference in the molecular mass between rib-glycated BSA (incubated for 7 days) and native BSA was 5,478 Da, showing ~41 rib (133 Da each) molecules bound to BSA. However, the retardation of BSA bands that had been incubated with other reducing sugars was not so distinct as that of BSA glycated with rib observed under the same conditions. No significant band retardation was exhibited with BSA alone or in the presence of suc which was used as a negative control. These results demonstrate that BSA is more vulnerable to rib glycation than to glycation with the other sugars tested.

**Figure 3:**
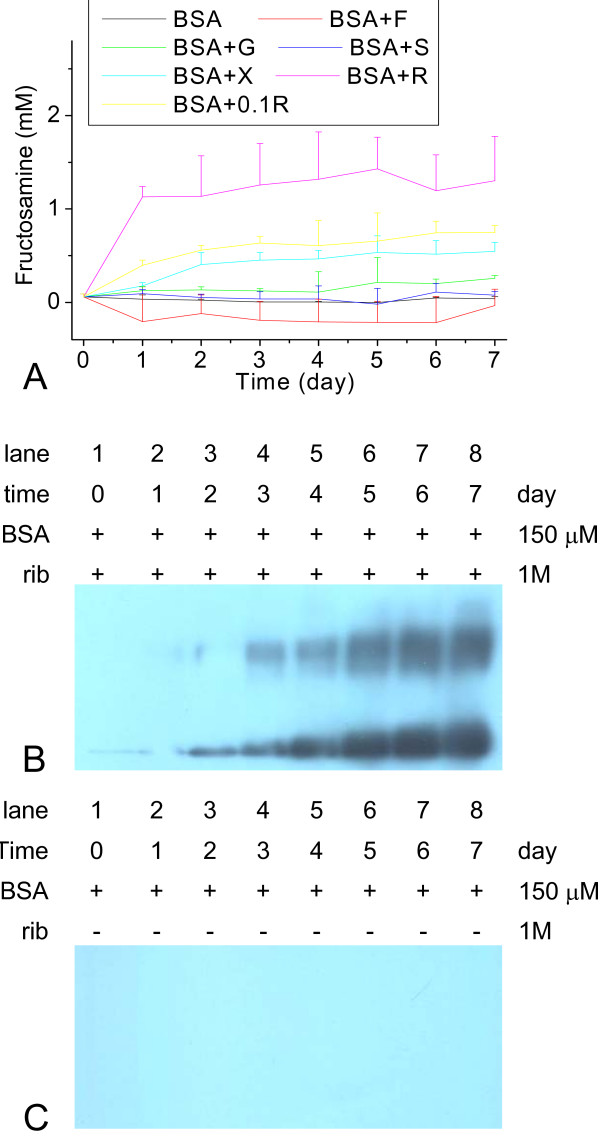
**Fructosamine assay of the products of BSA incubation with different saccharides and Western blots of D-ribose-glycated BSA**. Incubation conditions were as in Figure 1, except that aliquots were taken from incubation mixtures for NBT assays at different time intervals (A). Western blotting of D-rib-glycated BSA with anti-AGEs (B); and BSA in the absence of sugar (control) (C).

Formation of fructosamine during glycation of BSA with rib under experimental conditions, was evaluated using NBT assays, carried out as shown in Figure 3A. The concentration of fructosamine in the rib-glycated BSA solution increased markedly in the initial 24 hour (h) period, showing a more rapid glycation procedure for rib than other monosaccharides. This indicates again that rib is the most active sugar in the glycation of BSA under the experimental conditions used.

To confirm whether AGEs were formed during glycation with rib, a monoclonal antibody (anti-AGEs) which recognizes the N-(carboxymethyl) lysine protein adduct of a major immunological epitope in proteins modified with AGEs [32] was used in Western blotting. Results showed that AGEs formed at the initial stages of glycation, and increased markedly on the first day of BSA incubation with rib (Figure 3B). However, AGEs were not detected with BSA alone (Figure 3C) or glycated with the other reducing sugars under the same conditions (data not shown).

### Conformational changes of BSA during glycation in the presence of ribose

First, we attempted to detect whether the secondary structure of BSA was changed during glycation. However, no significant changes in CD spectra of the protein before and after glycation were detected. That is to say, both α-helix and β-sheet secondary structures (Additional file 1) were not markedly changed by glycation. It appears that changes in the conformation of rib-glycated BSA do not occur in secondary structures, but rather in higher order structures.

**Figure 4:**
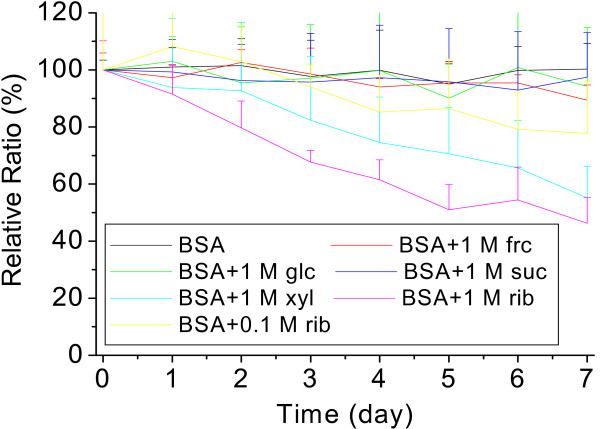
**Changes in the intrinsic fluorescence of BSA incubated with different saccharides**. Incubation conditions were as in Figure 1, except that aliquots were taken from incubation mixtures for measurements of the intrinsic fluorescence (λ_ex _280 nm; λ_em _335 nm) at different time intervals.

Intrinsic fluorescence is commonly used to study conformational changes of a protein in solution [33-35]. As shown in Figure 4, the intrinsic fluorescence of BSA decreased markedly during incubation with rib. Over the period of the 7 day incubation, the intensity of the intrinsic emission of rib-glycated BSA decreased by 50% compared with that of BSA alone as a control (100%). According to Swamy [36], a decrease in the intrinsic fluorescence results from exposure of tryptophanyl residues to solvent molecules that collide with fluorophores and consume the fluorescence energy. This suggests that the molecular conformation of rib-glycated BSA changes during glycation.

**Figure 5:**
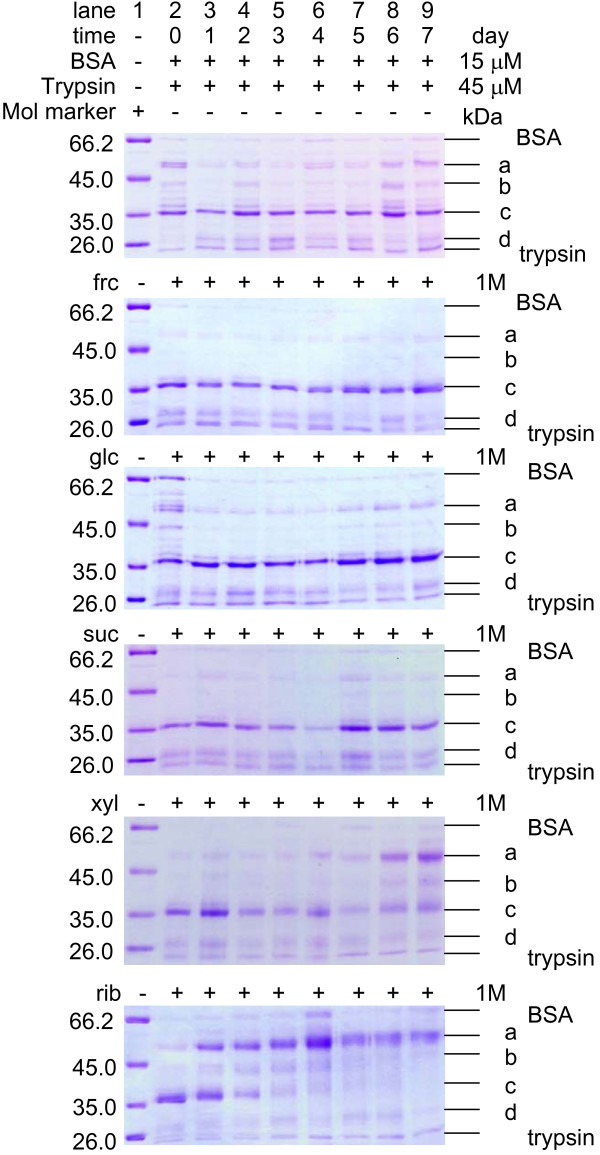
**Digestion of saccharide-treated BSA by trypsin**. Incubation conditions were as in Figure 1, except that aliquots were taken from the incubation mixtures at different time intervals for digestion by trypsin at the molecular ratio [BSA]/[trypsin] of 1:3 (37°C, 1 h).

Furthermore, we examined conformational changes of rib-glycated BSA using digestion with trypsin. We took aliquots at different time intervals during the glycation process for trypsin digestion (Figure 5). BSA (66 kDa) glycated with reducing monosaccharides released 4 common peptide fragments -*a*, -*b*, -*c*, and -*d *with apparent molecular masses of 57.0, 41.1, 36.7 and 27.2 kDa, respectively, in the presence of trypsin. Significantly, after incubation for 5 days, peptides -*a *and -*b *were not detected when rib-glycated BSA was incubated with trypsin. After incubation for 7 days, only peptide -*a *was observed. The four peptide fragments, however, were present on the SDS-PAGE for frc-, glc- and xyl-glycated BSA under the same conditions. In the presence of trypsin, BSA alone or with suc as a control released the same peptide fragments from day 1 to day 7. This suggests that the difference of the cleavage on BSA in the presence of trypsin arises from protein polymerization during glycation with rib. Maybe lys residues at the cleavage sites were blocked by glycation or the conformational rearrangement following increasing glycation by rib progressively uncovered the other cleavage sites while hindering accessibility to the a cleavage site.

### Globule aggregates of D-ribose-glycated BSA

**Figure 6:**
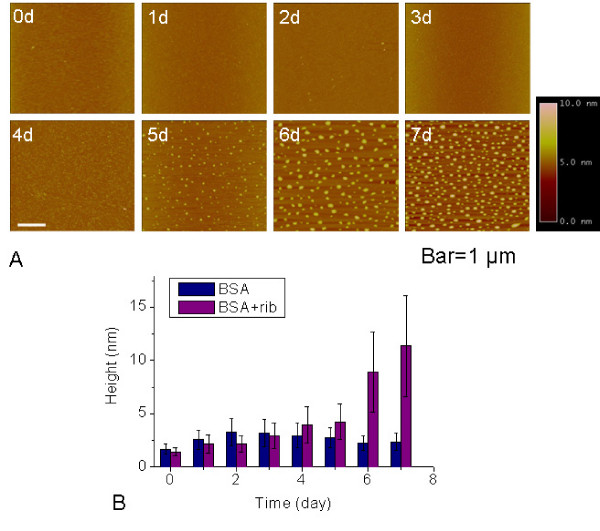
**Observation of BSA incubated with ribose by atomic force microscopy**. Incubation conditions were as in Figure 1, except that aliquots were taken from incubation mixtures at different time intervals for observation by AFM (A). The scale bar equals 1 μm. The horizontal diameter at half height was shown (B).

To clarify whether blockage of trypsin digestion resulted from protein polymerization, we employed atomic force microscopy (AFM) to observe particle size changes of rib-glycated BSA during glycation. As shown in Figure 6, the particle size of rib-glycated BSA started to increase from day 5 of the incubation period, exhibiting protein polymerization. A significant increase in particle size could be observed from day 6 of the incubation period. BSA glycated with the other monosaccharides as mentioned above showed no significant polymerization under these experimental conditions (Table 2). BSA alone (Additional file 2) or with suc as a control maintained constant particle size as determined by AFM. These results demonstrate that glycation with rib induces BSA to polymerize and form globular polymers.

**Table 2:** Diameter of BSA glycated with different monosaccharides.

Samples	Particle size (nm)	
	
	3 d	7 d
BSA+rib	4.146 ± 1.745	11.378 ± 4.758

BSA+suc	2.917 ± 0.882	3.028 ± 2.550

BSA+frc	3.010 ± 1.951	3.695 ± 2.184

BSA+glc	2.041 ± 1.412	2.481 ± 1.576

BSA+xyl	3.141 ± 2.278	3.521 ± 2.323

BSA	3.208 ± 1.257	2.385 ± 0.799

rib	1.434 ± 1.064	1.052 ± 0.696

**Figure 7:**
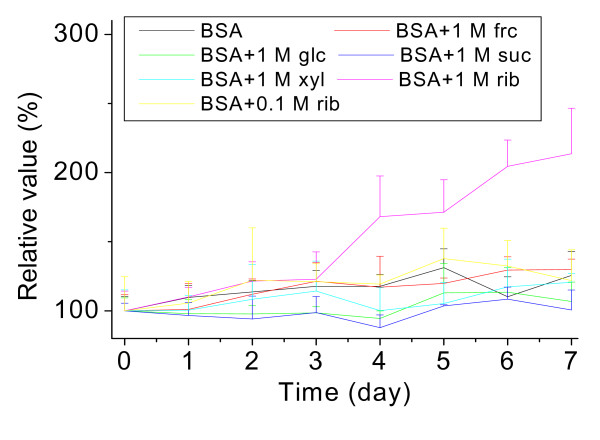
**Changes in the Thioflavin T fluorescence of BSA treated with different saccharides**. Thioflavin T (30 μM final concentration) was mixed with samples of BSA in different saccharide solutions for different time intervals. The fluorescence intensity of Thioflavin T was recorded (λ_ex _450 nm; λ_em _485 nm). Kinetics of the increase in fluorescence emission of ThT with glycated BSA are shown.

What are the characteristics of BSA polymers? To answer this question, we added Thioflavin T (ThT) a fluorescent reagent to test whether the polymers are amyloid-like aggregates (Figure 7). Fluorescence of ThT at 485 nm significantly increased in the presence of BSA incubated with rib for 4 days. Fluorescence intensity increased 170% relative to BSA alone as a control. However, BSA incubated with the other reducing monosaccharides frc, glc and xyl (including non-reducing sugar suc as a control) showed no significant changes in ThT fluorescence under our experimental conditions.

### Cytotoxicity of ribose-glycated BSA and its putative mechanism

**Figure 8:**
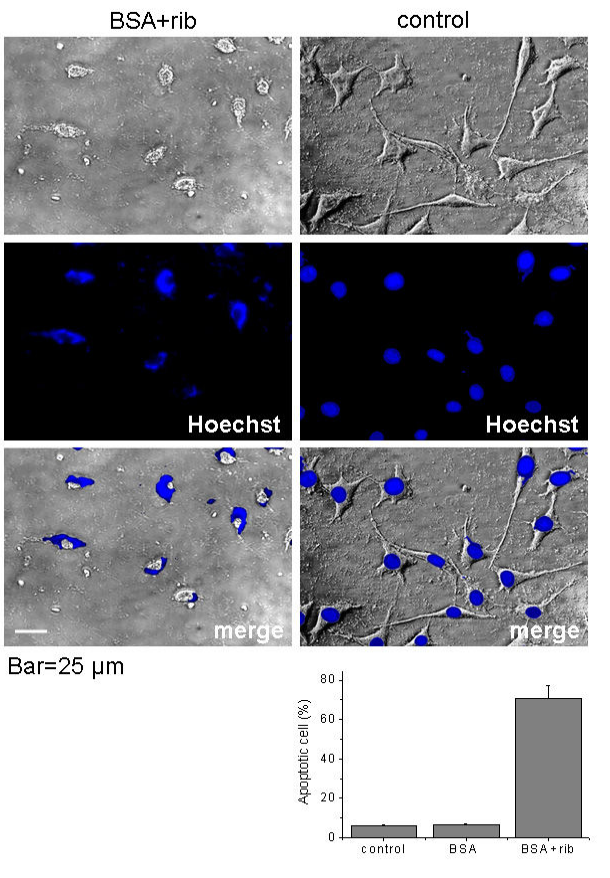
**Phase contrast microscope image of cells treated with glycated BSA**. SH-SY5Y cells were imaged after incubation with rib-glycated BSA (20 μM, 3-day sample) for 8 h. Normal cells were used as controls. Cells were visualized by inverted contrast microscopy. Statistics of apoptotic cells was also shown. Bar = 25 μm.

Having determined that rib-glycated BSA polymers were globular amyloid-like aggregates, we were concerned about the cytotoxicity of these aggregates, and thus tested the cytotoxicity of the glycated protein in cell culture. Morphological evaluation of cell line SH-SY5Y was carried out to investigate effect of rib-glycated BSA on neural cells. As shown in Figure 8, rib-glycated BSA markedly induced axonal atrophy, and cells became spherical with condensed nuclei, as visualized by Hoechst 33258 staining under fluorescence microscopy. BSA alone (or incubated with suc), and glycated BSA with the other reducing monosaccharides, did not show axonal atrophy and cell condensation under the experimental conditions used (data not shown). This suggests that rib-glycated BSA is strongly cytotoxic to neurons in cell culture.

**Figure 9:**
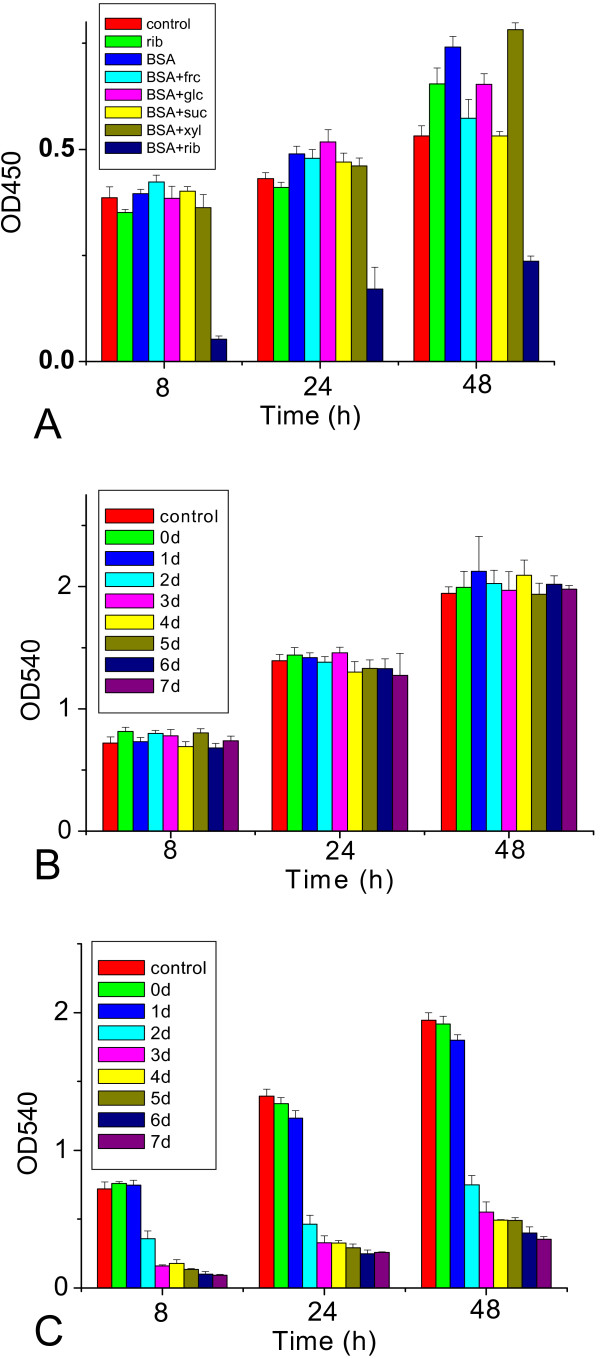
**Cell viability measured by CCK-8 and MTT assays**. BSA alone or incubated with different saccharides at 37°C for 3 days, was added to SH-SY5Y cells for 8 h and cell viability was measured using the CCK-8 assay at 8 h, 24 h, and 48 h after sample addition (A). BSA incubated with 0.1 M rib (B) or 1 M rib (C) for 0–7 days was added to SH-SY5Y cells for 8 h and cell viability was measured using the MTT assay at 8 h, 24 h, and 48 h after sample addition.

The effect of rib-glycated BSA on the viability of SH-SY5Y cells was then examined by using MTT and CCK-8 assays [37,38]. As shown in Figure 9A, the number of viable cells decreased significantly after incubation with rib-glycated BSA for 8 h, whereas a significant decrease in cell viability was not observed in the presence of BSA glycated with glc, frc and xyl or BSA alone. Moreover, decrease in cell viability induced by rib-glycated BSA occurred in a concentration-dependent manner; rib-glycated BSA formed after 3 or more days of incubation with rib showing cytotoxicity to cells exposed to rib-glycated BSA for at least 8 h (Figures 9B, C).

**Figure 10:**
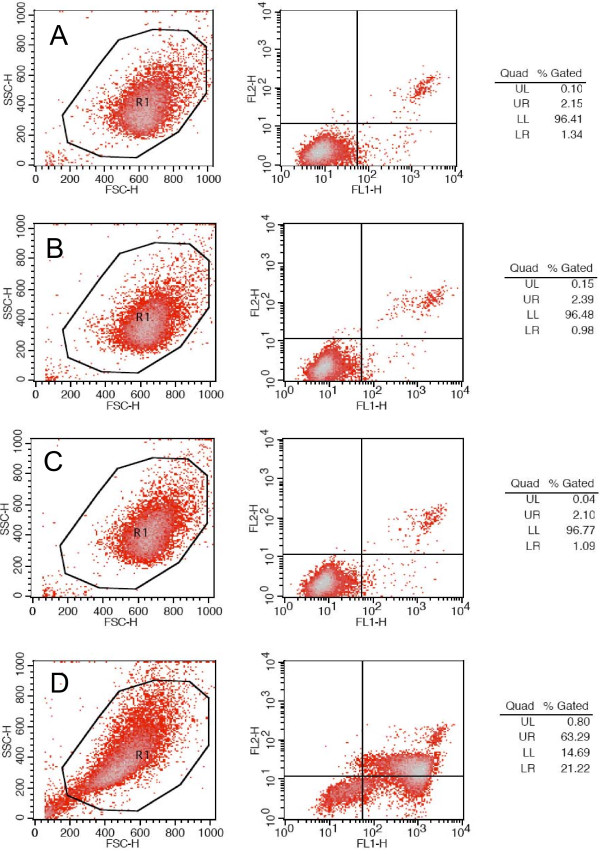
**Ribose-glycated BSA induces SH-SY5Y cell apoptosis**. Flow cytometric analysis of SH-SY5Y cells 24 h after treatment with rib-glycated BSA for 8 h (D). Normal cells (A), rib (B), BSA (C) were also shown.

In order to determine whether the neurotoxicity of rib-glycated BSA involved an apoptotic pathway, we used flow cytometry to examine the effects of rib-glycated BSA on SH-SY5Y cells (Figure 10). Cells that were incubated with rib-glycated BSA for 8 h and then treated with the dyes Annexin-V (AV, an indicator of apoptosis) and propidium iodide (PI, an RNA dye) [39] showed a clear increase in both LR (apoptotic) and UR (late apoptosis, necrosis) SH-SY5Y cell populations after 24 h. After 8 h of exposure to rib glycation conditions, there was a corresponding increase in cell apoptosis (+21.22%) and late apoptosis/necrosis (+63.29%) rates in comparison with untreated cells.

**Figure 11:**
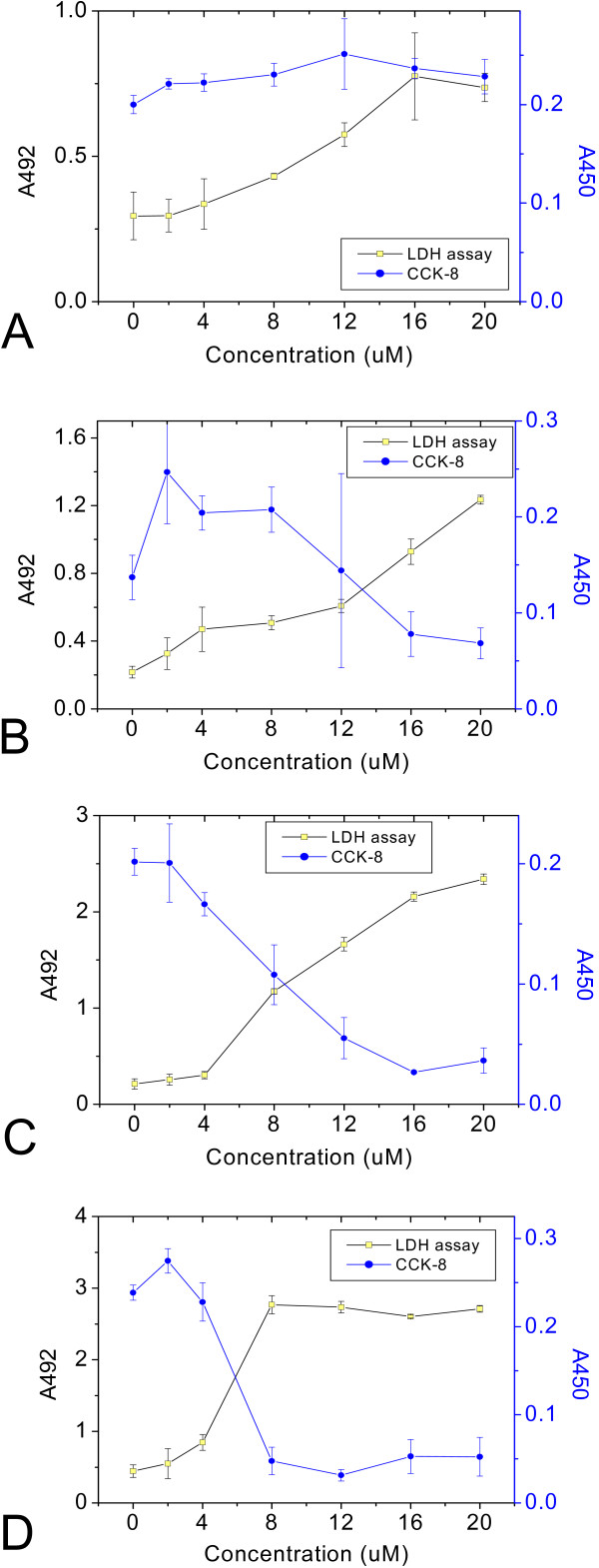
**Cytotoxicity detection of ribose-glycated BSA in SH-SY5Y cells**. Ribose-glycated BSA of different concentrations was added to SH-SY5Y cells. The LDH activity and CCK-8 assays were used to measure the cytotoxicity of samples after incubation for 4 h (A), 8 h (B), 12 h (C), and 24 h (D).

**Figure 12:**
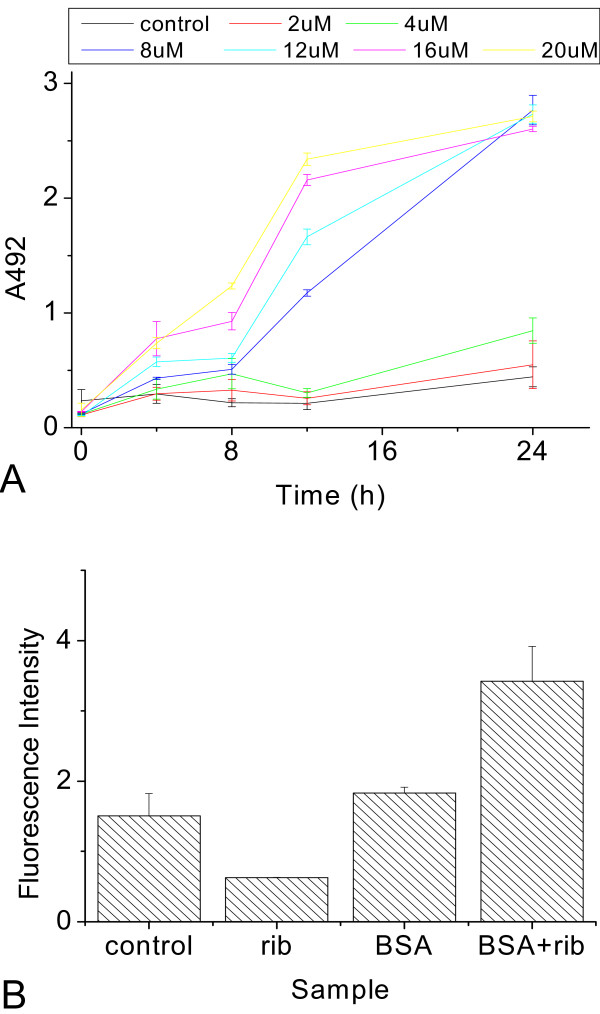
**LDH assay and measurement of ROS in SH-SY5Y**. Rib-glycated BSA of different concentrations was added to SH-SY5Y cells for different lengths of time, then LDH activity was measured using a cytotoxicity detection kit (Roche, Switzerland) (A). The ROS level of cells was measured 24 h after the addition of samples (B).

To further confirm that SH-SY5Y cells were damaged in the presence of rib-glycated BSA, an assay system for quantifying the release of LDH from cells was carried out as shown in Figures 11 and 12A. Ribose-glycated BSA at concentrations of 5–8 μM triggered dose-dependent cytotoxicity of cells incubated for 8 h. However, cytotoxicity of BSA alone or glycated with the other reducing sugars was not detected under the same conditions (data not shown). The significant increase in plasma membrane permeability detected by LDH assay indicates damage of SH-SY5Y cells exposed to rib-glycated BSA.

To clarify whether free radicals had a role in causing lesions in SH-SY5Y cells exposed to rib-glycated BSA, we measured intracellular levels of ROS. As shown in Figure 12B, the intracellular level of ROS increased significantly when cells were exposed to rib-glycated protein compared with rib or BSA alone. This suggests that the cell death induced by globular amyloid-like aggregation of rib-glycated BSA is related to the intracellular ROS pathway.

## Discussion

Glycation is a stepwise process that begins with a nucleophilic addition reaction between a free amino group of a protein and a carbonyl group from a reducing sugar, forming a reversible intermediate product (Schiff's base). The Schiff's base can turn into a stable ketoamine by an Amadori rearrangement [1]. The next step is the formation of numerous intermediary products, some of which are very reactive. The final step consists of crosslink formation between products in which heterogeneous structures called advanced glycation end products (AGEs) are formed [40]. Transformation of Schiff's bases to relatively stable yet reversible ketoamines is slow and requires a long time [11,41], but AGEs are formed rapidly from ketoamines [13,42]. In this work, as can be seen in Figure 3, the glycation of BSA with rib results in rapid production of AGEs, almost within 24 h under the experimental conditions used. The speed of conversion from Schiff's bases to stable ketoamines for BSA exposed to rib during glycation was much faster than for BSA exposed to glc. That is to say, the glycation speed of rib with BSA was the most rapid of the reducing sugars used in this work.

NBT assays, changes in fluorescence (410 nm and 425 nm) and protein intrinsic fluorescence (335 nm) showed that the furanoses rib and xyl are much more active in the glycation of BSA than the pyranoses glc and frc. Of the five sugars, rib is the most active. Glc exists in solution as an intramolecular hemiacetal in which the free hydroxyl group at C-5 has reacted with the aldehydic C-1, rendering the latter asymmetric and giving rise to stereoisomers. The six-membered aldopyranose ring is much more stable than the aldofuranose five-membered ring because the pentose ring for rib and xyl is not planar but occurs in one of a variety of conformations generally described as "puckered" [43]. The unstable aldofuranose ring is vulnerable to reactions with amino groups. This may be one reason why furanoses are more reactive in glycation than pyranoses. The difference in glycation activity of rib and xyl is probably due to different configurations of the hydroxyl group at C-3.

BSA contains two tryptophanyl residues (Trp 135 and Trp 214) that are largely responsible for the protein intrinsic fluorescence (335 nm). As shown in Figures 1 and 4, intrinsic fluorescence decreases markedly while emission at 410 nm and 425 nm increases as incubation period lengthens. Fluorescence at 410 nm and 425 nm reflects the formation of AGEs, and thus it is reasonable for rib-glycated BSA to show a marked increase in emission intensity [44]. The decrease in intrinsic fluorescence is related to a conformational change in BSA. This results from exposure of Trp 135 and Trp 214 residues to solvent molecules which have higher colliding frequency with exposed tryptophanyl residues during glycation. This suggests that the molecular conformation of BSA becomes more random during its reaction with rib.

Changes observed in fluorescence at 410 nm and 425 nm and intrinsic fluorescence (335 nm) are somewhat different to results observed from Western blotting with AGE-antibodies. As shown in Table 1, the rate of formation of AGEs from rib-glycated BSA was significantly faster than that detected by fluorescence. Anti-AGEs recognize N^$^-carboxymethyllysine(CML)-protein adducts as epitopes [32,45]. Fluorescence, however, is not only related to the characteristic of the fluorophore (the formed glycation product), but also to protein conformational change. The image of rib-glycated BSA can be seen on the mica surface under AFM (Figure 6A). Formation of rib-glycated BSA polymers occurred from day 5 of the incubation period. That is to say, protein conformational changes followed after the formation of AGEs from rib-glycated BSA.

CD spectra did not show distinct changes after glycation of BSA with rib (Additional File 1), i.e. secondary structures of rib-glycated BSA did not change relative to control native BSA. Results from fluorescence observations showed that BSA did undergo changes in molecular conformation. Although changes in Trp fluorescence are strictly symptomatic of local changes in the tryptophanyl residue microenvironment, they are usually also associated with larger structural rearrangements of the peptide chains [46]. This suggests that conformational changes that occurred were protein polymerization under the experimental conditions. This theory was confirmed using AFM. BSA molecules polymerized into globular aggregates in the presence of rib from day 5 of the incubation period (Figure 6). According to Sattarahmady, glycation leads to formation of molten protein globules [19]. We hypothesize that molten globules are probably prefibrils with some characteristic of amyloid-like aggregations.

To investigate whether rib-glycated BSA forms amyloid-like aggregations, we measured the fluorescence at 485 nm of ThT incubated with the glycated product (Figure 7), as ThT fluorescence is commonly used to detect the formation of amyloid-like aggregations [46-51]. Our results show that emission intensity was markedly increased during the rib-glycation process. According to recent studies, ThT has been found to bind to proteins in the nonfibrillar state (oligomers), probably because of the open structure of these proteins. Some protein molten globules are positive in ThT fluorescence [46,49]. Thus, we thought that polymers of rib-glycated BSA are present as amyloid-like aggregations. In other words, ribosylated BSA is probably in molten globules with amyloid-like characteristics. On this point, fibrils might arise when BSA is exposed to rib for a longer time, which needs further investigation.

Globular-like amyloid protein aggregations are known to have fatal neurotoxicity [52]. Previous work in our lab showed that the globular-like amyloid tau protein induced by formaldehyde has significant cytotoxicity to SH-SY5Y cells and HEK-293 cells [53-55]. Western blotting results in Figure 3 show that the toxicity of rib-glycated BSA is related to AGEs. Xyl-glycated BSA did not show significant cytotoxicity to SH-SY5Y cells because little AGEs were formed from xyl-glycated products under these experimental conditions, i.e. it is AGEs or intermediate products of rib-glycated BSA that act as cytotoxic agents to induce SH-SY5Y cell death. Thus, rib-AGEs were used rather than rib-glycated BSA in following tests.

As illustrated in Figures 9 and 11, exposure to rib-AGEs inhibited SH-SY5Y cell viability and induced cytotoxity. As described by Yamagishi and colleagues [56], AGEs arising from glc-glycated proteins lead to cell death via apoptosis. Results from LDH assays (Figure 12A) showed that the LD_50 _of rib-glycated BSA for SH-SY5Y cells was approximately 5 μM, similar to that obtained for the amyloid-like tau protein (10 μM) [54]. On the basis of the following observations we conclude that rib-glycated BSA induced SH-SY5Y cell death via an apoptotic process. (1) SH-SY5Y cells showed neurite atrophy and became spherical with condensed nuclei in the presence of rib-AGEs. (2) CCK-8, MTT and LDH assays indicated that rib-AGEs decreased the viability of SH-SY5Y cells and induced cell cytotoxicity. (3) flow cytometry analysis of Annexin V/PI confirmed that apoptosis was induced by rib-AGEs.

**Figure 13:**
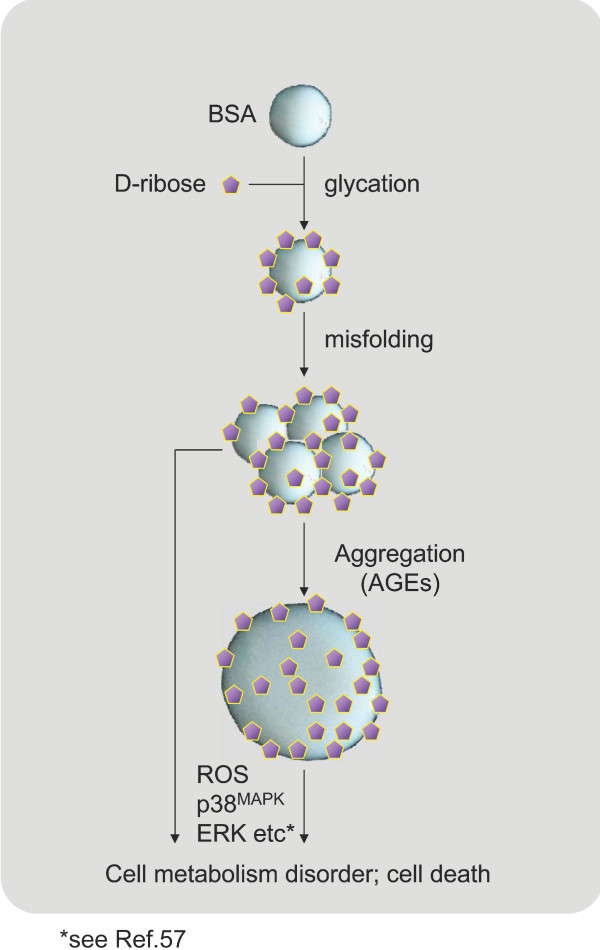
**Putative mechanism of apoptosis of SH-SY5Y cells induced by rib-glycated BSA**. Glycation of BSA with rib induces protein misfolding and results in the formation of amyloid-like aggregations with high cytotoxicity that trigger cell death by the activation of cellular signalling cascades.

Exposure to rib-AGEs induced a marked increase in intracellular levels of ROS in SH-SY5Y cells (Figure 12B). This suggests a correlation between exposure to rib-AGEs and production of ROS in the cells. Here we propose a putative mechanism (Figure 13) for the apoptosis of SH-SY5Y cells induced by rib-glycated BSA. Glycation of BSA with rib induces protein misfolding and results in the formation of amyloid-like aggregations (molten globules) with high cytotoxicity that trigger cell death by the activation of cellular signalling cascades [57].

A number of issues require further explanation. Firstly glycated BSA was prepared using a high concentration (1 M) of reducing monosaccharides. This is common to protocols used in many laboratories [13,17,19,44]. At such a high concentration of reducing sugars, it is possible to clarify which monosaccharide is most active in protein glycation and has the strongest cytotoxicity. Here we have established a useful model for preparing rib-glycated proteins which can be used for preparing many other glycated proteins readily, rapidly and repeatedly in the presence of rib under these experimental conditions.

Secondly, rib is found in all living cells and on average, the body contains about 16 mg of rib per litre of blood (~100 μM), i.e. a roughly ten-fold lower level than that of blood glc. Ribose exists in the cerebrospinal fluid and Seuffer has determined the concentration (0.01–0.1 mM) [58]. The level of rib-glycated protein in blood is still unknown because of its relatively low concentration in human blood. The amount of glc-glycated albumin and hemoglobin products in the blood is known, but so far there is no successful method for identifying whether glycated proteins are coupled with rib or glc in blood. However, rib-glycated BSA is strongly toxic to neuroblast cells, with a very low LD_50 _(~5 μM glycated BSA). The level of rib in the blood (~100 μM) is about 20 times higher than the LD_50 _of its glycated protein. Although complications resulting from the rib-glycated protein have not yet been clarified, attention should be paid to the effects of blood rib when the level is markedly elevated.

Thirdly, as mentioned above, rib-glycated BSA was the most toxic of the pyranose- or furanose-glycated BSA tested to human neuroblast cells (SH-SY5Y). Ribose is of vital importance to many activities of the cell and provides a backbone for synthesising the most important molecules in the body, such as ATP, the "energy currency" all cells need to function normally. In humans, it is synthesized from glc and can also be used to make glc. This monosaccharide is active and rapidly consumed. Thus far, no evidence of complications resulting from rib-glycation have been presented. In fact, rib has been used as a nutrient energy supply.

Finally, as shown in further experiments (unpublished data), rib-glycated BSA is not only toxic to SH-SY5Y cells, but is also toxic to BV2, a glia cell line. Ribose exists both within and outside cells, and thus this monosaccharide has the opportunity to glycate both intracellular and extracellular proteins. The glycated products may be harmful to cells and lead to damage of some important cell types such as neurons and glia in the central nerve system. For this reason, several native proteins were reported to protect nerve cells from amyloid cytotoxicity, for example, butyrylcholinesterase can inversely associate with the soluble Aβ conformers and delay the onset and decrease the rate of Aβ fibril formation *in vitro *[59]. However, whether rib-glycation is related to complications such as neurodegeneration requires further investigation.

## Conclusion

Here we investigated the effect of D-ribose on protein misfolding and aggregation. Compared with other monosaccharides, D-ribose is rapid in glycating BSA, and can induce protein misfolding and aggregation, as observed by fluorescence measurements, leading to the formation of amyloid-like deposits that appeared as densely staining granules under atomic force microscopy, and bound the amyloid-specific dye thioflavin T. The amyloid-like aggregates (molten globules) of BSA were observed to induce apoptosis in neurotypic SH-SY5Y cells as stained by Hoechst 33258, MTT and CCK-8 assay, LDH activity assay, and flow cytometry using Annexin V and Propidium Iodide staining, as well as ROS measurements. These results suggest that D-ribose may play an important role in AGE-related diseases.

## Methods

### Materials

Reducing monosaccharides employed in this work such as pyranoses (glc, frc), furanoses (rib and xyl), non-reducing disaccharides suc, and trypsin were from Amresco (USA). Bovine serum albumin (BSA) and thioflavine T came from Sigma (USA) and Aldrich (USA). Other chemicals were of analytical grade.

### Glycation of BSA

#### a. Preparation

BSA was dissolved in 20 mM Tris-HCl (pH 7.4) to yield a stock solution of 20 mg/ml. This solution was then resuspended with rib prepared in Tris-HCl (pH 7.4) to a final concentration of 10 mg/ml BSA and 0.1 M or 1 M monosaccharide. BSA alone and in the presence of frc, glc, xyl or suc was used as a control. Reaction mixtures were incubated at 37°C for 0 to 7 days. All solutions were filtered with 0.22 μm membranes (Millipore, USA).

#### b. SDS-PAGE

Aliquots of glycated protein samples were subjected to SDS-PAGE. For the digestion experiment, BSA (final conc. 0.5 mg/ml) and trypsin (final conc. 0.5 mg/ml) were mixed in Tris-HCl buffer (pH 7.4) to give a volume of 100 μl, and incubated at 37°C for 1 h. Aliquots were subjected to electrophoresis using Bio-Rad (USA) electrophoresis equipment.

#### c. Nitroblue tetrazolium (NBT) assay

For NBT assay, we followed a published method [25,60] with the following modifications. 200 μl of 0.75 mM NBT (Amresco, USA) was added to a 96-well microplate along with 10 μl of the unknown sample or standard. The kinetics for reduction of the dye by fructosamine groups (0.1 M carbonate buffer, pH 10.35) was measured at 540 nm using a MK3 microplate reader (Thermo, USA) after incubating for 30 min at 37°C. Standard curves were generated by addition of 10 μl of 1-deoxy-1-morpholino-D-fructose (1-DMF, Sigma, USA). Fructosamine formation was monitored by comparison to standard curves (R^2 ^> 0.99).

#### d. Western blotting

Aliquots of BSA incubated with rib for different durations were subjected to electrophoresis. The proteins were then transferred onto PVDF membranes, and probed with anti-AGEs (dilution = 1:1000, 6D12, Wako, Osaka, Japan) followed by goat anti-mouse horseradish peroxidase (HRP) (KPL, Gaithersburg, Maryland, USA) at a dilution of 1:2000. Immunoreactive bands were visualized using enhanced chemiluminescence (Pierce, USA).

### Fluorescence measurements

Fluorescence of the advanced glycated end-products was monitored on an F-4500 fluorophotometer (Hitachi, Japan). Wavelengths (λ_ex_370 nm/λ_em_425 nm; λ_ex_320 nm/λ_em_410 nm) were employed [24]. Desired final protein concentration was 0.1 mg/ml (1.5 μM). Protein intrinsic fluorescence at 335 nm was also measured by excitation at 280 nm. Thioflavin T (ThT, 30 μM), commonly used to detect protein aggregations, was added to glycated BSA (1.5 μM) to investigate whether any amyloid-like deposits formed at 37°C. The fluorescence of ThT (λ_ex_450 nm/λ_em_485 nm) was measured at 25°C with a bandwidth of 1.5 nm. Changes in emission intensity (%) per day are presented as ratios.

### Circular dichroism (CD) spectropolarimetry measurements

CD spectra were recorded using a Jasco J-720 CD spectrometer (Japan). The spectra were measured in 1 mm pathlengths of a quartz cuvette, and data were scanned from 190 nm to 260 nm at 1 nm intervals. The final protein concentration was 1 mg/ml (15 μM). The bandwidth was set at 1 nm (25°C). Baselines of the spectra were calibrated using the spectrum of the buffer measured under identical conditions. All experiments were repeated 10 times and averaged. The background of the corresponding buffers without protein or rib was subtracted for all samples.

### Atomic Force Microscope (AFM) measurements

Protein samples were diluted using Tris-HCl buffer (pH 7.4) and 10 μl of BSA (10 μg) was dropped onto the mica surface and left for 5 min at room temperature before drying with nitrogen gas. The mica diaphragm was rinsed 20 times with ultrapurified water and dried with nitrogen gas before observation under the atomic force microscope (Mutiplemode-I, Digital Instruments, USA). The horizontal diameter at half height of a particle (globular protein) was measured and data were analyzed using Nanoscope 6.11r1 software (USA).

### Cell culture

SH-SY5Y cells were cultured in Dulbecco's modified Eagle's medium supplemented with 100 IU/ml penicillin and 100 μg/ml streptomycin at 37°C in a humidified 5% CO_2 _incubator as described [61]. The medium contained 10% fetal bovine serum. Cells were grown to 70–80% confluence in 25 mm diameter dishes and fed every fourth day. For all experiments, the culture medium was replaced with serum-free medium before the addition of the glycated protein. Cells were incubated with the samples (glycated BSA with each monosaccharide) (20 μM) for 8 h. After that, medium was changed to DMEM containing 10% fetal bovine serum.

### Cell viability test

As described by Mayo and Tominaga [37,38], viability was assessed using the 3-(4, 5-dimethylthiazol-2-yl)-2,5-diphenyl tetrazolium bromide (MTT) test or cell counting kit-8 (CCK-8). MTT and CCK-8 were from Beyotime (China).

### a. MTT assay

SH-SY5Y cells were seeded on a 96-well plate at a concentration of 10^5^cells per well and either exposed or not-exposed to the glycated protein (20 μM) for 8 h. The culture media was then changed to DMEM with 10% fetal bovine serum. 50 μl MTT (final concentration 0.5 mg/ml) were added at 8, 24, and 48 h, respectively, after adding the glycated protein. Plates were incubated at 37°C for 4 h, and then the assay was stopped by replacement of the MTT-containing medium with 150 μl dimethysulfoxide (DMSO) and the absorbance at 540 nm was recorded. Absorbance measurements were recorded using a Multiscan Mk3 (Thermo Electron Corporation, USA).

### b. CCK-8 assay

SH-SY5Y cells were seeded on a 96-well plate at a concentration of 10^5^cells per well and either exposed or not-exposed to the glycated protein (20 μM) for 8 h. The culture media was then changed to DMEM with 10% fetal bovine serum. The CCK-8 reagent were added at 8, 24, and 48 h, respectively, after adding the glycated protein. Plates were incubated at 37°C for 1 h and the absorbance at 450 nm was recorded.

### Cytotoxicity Detection

LDH cytotoxicity assays were performed according to the manufacturer's protocol (Roche, Switzerland). This colorimetric assay quantifies activity of LDH released from the cytosol of damaged cells into the supernatant and thus serves to quantify cell death [62,63].

### Measurement of intracellular ROS

The level of cytosolic ROS was measured by DCFH-DA (Beyotime, China) as described [64]. Briefly, SH-SY5Y cells were grown in a 24-well plate and incubated with rib, BSA, and rib-glycated BSA for 8 h. Normal cells were used as controls. Cells were washed with PBS and incubated with DCFH-DA for 30 min. DCFH-DA was initially non-fluorescent and was converted by oxidation to the fluorescent molecule DCFH (λ_ex_485 nm/λ_em_538 nm). DCFH was then quantified using a CytoFluor Multi-well Plate Reader (Fluoroskan Ascent, Thermo Lab Systerms, USA).

### Flow cytometric analysis

Cells undergoing apoptosis were detected by double staining with Annexin V-FITC/PI in the dark according to the manufacturer's instructions [39]. Cells attached to Petri dishes were harvested with 0.25% trypsin and washed twice with cold PBS. Cell pellets were suspended in 1× binding buffer (10 mM HEPES/NaOH, pH 7.4, 140 mM NaCl, 2.5 mM CaCl_2_) at a concentration of 1 × 10^6 ^cells/ml. Then the cells were incubated with Annexin V-FITC and propidium iodide (PI) for 15 min (22–25°C) in the dark. The stained cells were immediately analyzed by flow cytometry (FAC Svantage SE, USA). Each measurement was carried out in at least triplicate.

## Authors' contributions

YW carried out the SDS-PAGE and cell experiments and drafted the manuscript. LC designed the experiments and carried out the NBT and Western Blotting experiments. JC carried out fluorescence measurements, CD, and AFM experiments. LG participated in AFM experiments. RQH conceived the study, participated in its design and coordination and helped to draft the manuscript. All authors read and approved the final manuscript.

## Supplementary Material

Additional file 1 **Changes in CD spectra of BSA in the presence of D-ribose.** Incubation conditions were as in Figure 1, except that aliquots of the incubation mixtures were taken for measurement of the CD spectra at different time intervals. BSA (A), BSA with rib (B). Click here for file

Additional file 2 **Observation of BSA by atomic force microscopy.** Incubation conditions were as in Figure 6A, except that BSA alone was observed rather than BSA incubated with rib. Click here for file
