# Supplementary figures and images for: Rapid glycation with D-ribose induces globular amyloid-like aggregations of BSA with high cytotoxicity to SH-SY5Y cells
Source: BMC Cell Biol. 2009 Feb 13;10:10. doi: 10.1186/1471-2121-10-10 (PMC2656460; doi:10.1186/1471-2121-10-10)

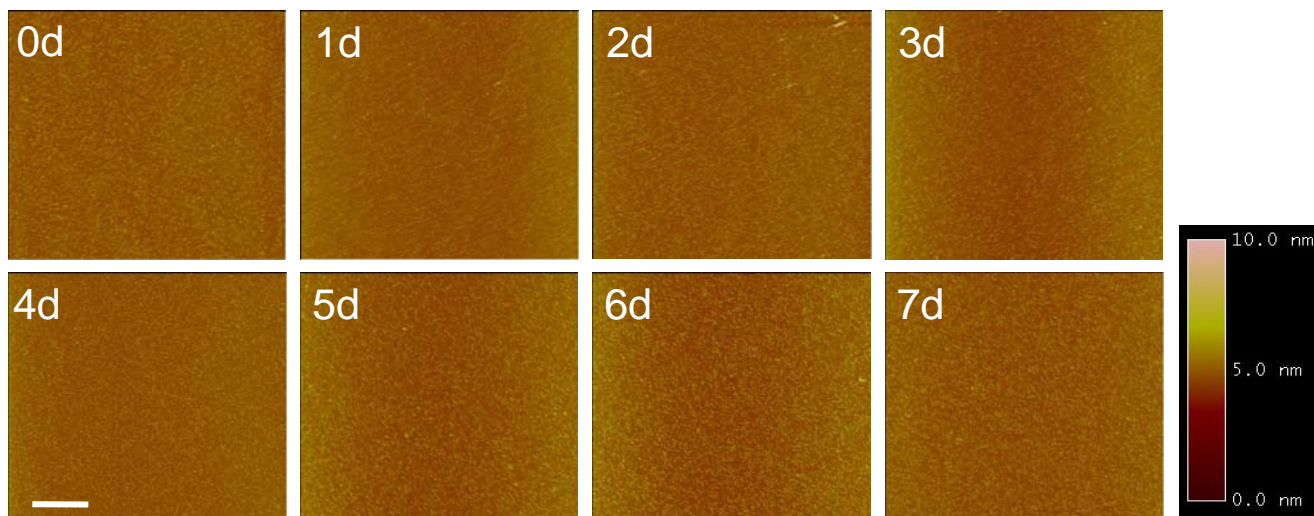

Bar=1  $\mu\text{m}$

Supplement: Additional file 2 — Observation of BSA by atomic force microscopy. Incubation conditions were as in Figure 6A, except that BSA alone was observed rather than BSA incubated with rib. [file 1471-2121-10-10-S2.pdf]
