# Supplementary material for: Rapid glycation with D-ribose induces globular amyloid-like aggregations of BSA with high cytotoxicity to SH-SY5Y cells
Source: BMC Cell Biol. 2009 Feb 13;10:10. doi: 10.1186/1471-2121-10-10 (PMC2656460; doi:10.1186/1471-2121-10-10)
Supplement: Additional file 1 — Changes in CD spectra of BSA in the presence of D-ribose. Incubation conditions were as in Figure 1, except that aliquots of the incubation mixtures were taken for measurement of the CD spectra at different time intervals. BSA (A), BSA with rib (B). [file 1471-2121-10-10-S1.pdf]

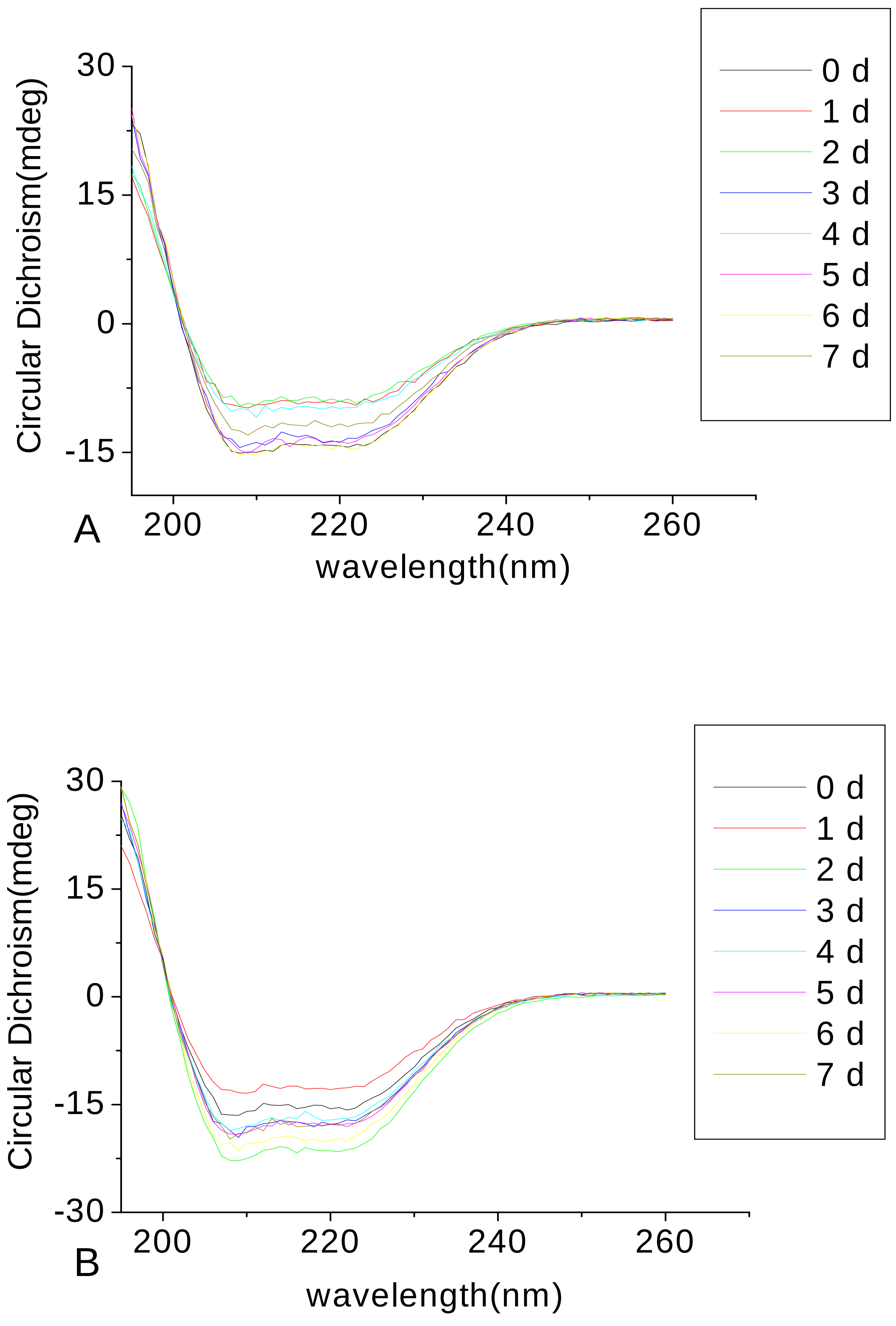
Circular Dichroism(mdeg)
30
15
0
-15
200
220
240
260
wavelength(nm)
0 d
1 d
2 d
3 d
4 d
5 d
6 d
7 d
A
Circular Dichroism(mdeg)
30
15
0
-15
-30
200
220
240
260
wavelength(nm)
0 d
1 d
2 d
3 d
4 d
5 d
6 d
7 d
B
